# MicraAV Versus Dual-Chamber Transvenous Pacemakers in Patients with Atrioventricular Block Following Transcatheter Aortic Valve Replacement: A 12-Month Single-Center Retrospective Study

**DOI:** 10.3390/jcm15207831

**Published:** 2026-10-10

**Authors:** Hiroshi Fukunaga, Kenichi Sasaki, Akira Kasagawa, Daisuke Togashi, Toshiya Yoshida, Ikutaro Nakajima, Shingo Kuwata, Masaki Izumo, Tomoo Harada, Yoshihiro J. Akashi

**Affiliations:** Department of Cardiology, St. Marianna University School of Medicine, 2-16-1 Sugao, Miyamae-ku, Kawasaki 216-8551, Kanagawa, Japan; hfukuna@shi.heart.or.jp (H.F.); a2kasagawa@marianna-u.ac.jp (A.K.); daisuke.togashi@marianna-u.ac.jp (D.T.); toshi.rasty236@marianna-u.ac.jp (T.Y.); ikutaro0817@marianna-u.ac.jp (I.N.); s2kuwata@marianna-u.ac.jp (S.K.); m2izumo@marianna-u.ac.jp (M.I.); tomoo-harada-md@marianna-u.ac.jp (T.H.); johnny@marianna-u.ac.jp (Y.J.A.)

**Keywords:** AV synchronous pacing, heart failure, leadless pacemaker, Micra, TAVR

## Abstract

**Background:** Data directly comparing the atrioventricular (AV)-synchronous leadless pacemaker (MicraAV) with a dual-chamber transvenous pacemaker (DDD) after transcatheter aortic valve replacement (TAVR) remain limited. **Methods:** We retrospectively studied patients undergoing pacemaker implantation for advanced or complete AV block within one month after TAVR at a single Japanese center between 2016 and 2025. After exclusions, the primary cohort comprised 74 patients (MicraAV, *n* = 29; DDD, *n* = 45). Clinical outcomes were assessed through 12 months, with death treated as a competing event for nonfatal outcomes. Device and echocardiographic parameters were evaluated longitudinally. **Results:** MicraAV recipients were less frequently female than DDD recipients (48.3% vs. 75.6%, *p* = 0.025). In the MicraAV and DDD groups, respectively, all-cause mortality was 6.9% and 6.7% (unadjusted HR, 1.12; 95% CI, 0.19–6.68; *p* = 0.904), heart failure rehospitalization occurred in 17.2% and 4.4% (subdistribution HR, 4.44; 95% CI, 0.86–22.92; *p* = 0.075), and device-related complications occurred in 6.9% and 4.4% (subdistribution HR, 1.60; 95% CI, 0.23–11.40; *p* = 0.636). The median MicraAV tracking index was 88.4% [IQR, 66.4–95.1]. No significant group-by-time interactions were observed for device electrical performance or echocardiographic left ventricular parameters. Tricuspid regurgitation worsened over time in the MicraAV group. **Conclusions:** All-cause mortality and device-related complications were infrequent in both groups, whereas heart failure rehospitalization occurred more frequently in the MicraAV group. Given the small number of events and wide confidence intervals, these findings should be considered exploratory and require confirmation in larger contemporary cohorts.

## 1. Introduction

Despite advances in valve design and implantation technique, conduction disturbances are still among the most common complications of transcatheter aortic valve replacement (TAVR) and frequently necessitate permanent pacemaker implantation [1,2]. For atrioventricular (AV) conduction disorders, including advanced or complete AV block, dual-chamber transvenous pacemakers (DDD) have traditionally been the standard of care, as they preserve AV synchrony. Leadless pacemakers, by contrast, were initially developed for select patients with permanent atrial fibrillation and bradycardia, limited venous access, or a heightened risk of device-related infection [3]. Following their introduction, however, leadless pacemakers have been widely adopted in the TAVR setting owing to their minimally invasive nature and favorable safety profile, attributes that may be particularly advantageous in the elderly and frail populations typically undergoing TAVR [4].

The first-generation leadless pacemaker (MicraVR; Medtronic, Minneapolis, MN, USA) provides ventricular-only pacing and therefore does not preserve AV synchrony. Prior studies have reported higher rates of heart failure hospitalization among patients with high-degree AV block or sinus node dysfunction treated with MicraVR compared with those receiving DDD [5,6]. The second-generation device (MicraAV; Medtronic), which incorporates atrial mechanical sensing to facilitate AV-synchronous ventricular pacing, has largely addressed this limitation and broadened the potential role of leadless pacing in patients with AV conduction disturbances [7].

Although previous studies have compared leadless and transvenous pacemakers after TAVR [4,8], evidence specifically comparing MicraAV with DDD remains limited. This distinction is clinically relevant because MicraAV, unlike ventricular-only leadless pacemakers, uses atrial mechanical sensing to facilitate AV-synchronous ventricular pacing. Detailed data comparing clinical outcomes, pacing characteristics, atrial mechanical tracking, and longitudinal echocardiographic changes between Micra AV and DDD after TAVR are scarce. Therefore, we sought to compare clinical outcomes and longitudinal device and echocardiographic parameters between Micra AV and DDD in patients requiring permanent pacing for AV conduction disturbance after TAVR.

## 2. Materials and Methods

### 2.1. Study Population

**Figure 1:**
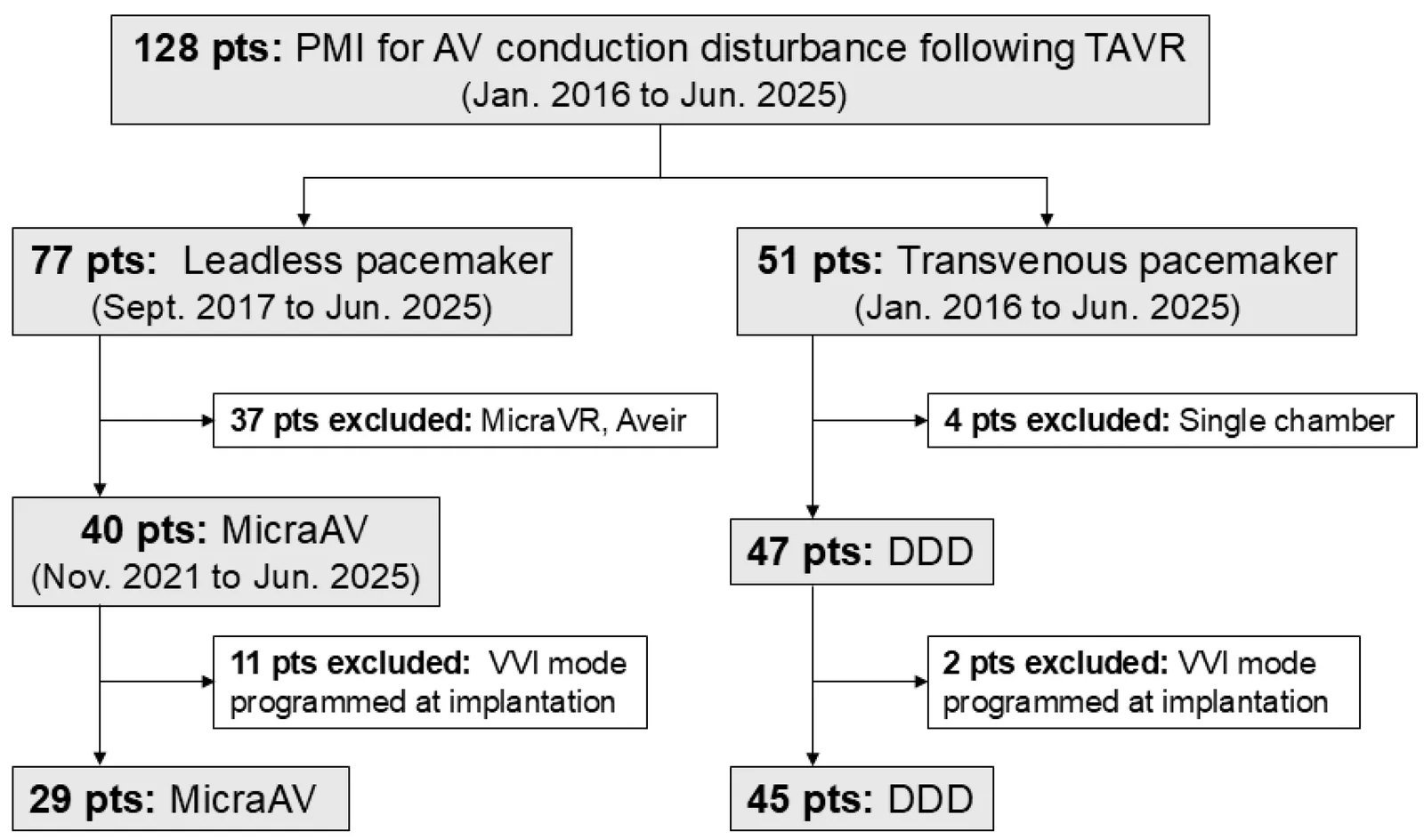
Flow chart of study population. AV: atrioventricular, DDD: dual-chamber transvenous pacemakers, PMI: pacemaker implantation, pts: patients, TAVR: transcatheter aortic valve replacement.

We retrospectively screened consecutive patients who underwent TAVR at St. Marianna University School of Medicine Hospital (Kawasaki, Japan) from January 2016 through June 2025. Patients were eligible for this study if advanced or complete AV block required permanent pacemaker implantation within one month after TAVR. Of 128 screened patients, those who received MicraVR, another leadless pacemaker (Aveir; Abbott, Abbott Park, IL, USA), a single-chamber transvenous pacemaker, or a device programmed to VVI mode at the time of implantation were excluded from the primary analysis. Among patients receiving MicraAV or DDD, VVI programming at implantation was selected when the anticipated ventricular pacing burden was low and pacing was intended primarily as backup support rather than continuous AV-synchronous pacing. The final primary cohort comprised 74 patients with AV-synchronous pacing: 29 treated with MicraAV and 45 with DDD (Figure 1). Clinical, device-related, and echocardiographic data were retrospectively collected and analyzed.

All patients provided written informed consent prior to pacemaker implantation. This study adhered to the principles of the Declaration of Helsinki, and the protocol received approval from the St. Marianna University School of Medicine Institutional Review Board (approval number: 6980). Informed consent for the use of clinical data for research purposes was obtained via an opt-out procedure.

### 2.2. TAVR Procedure

Eligibility for TAVR was determined through multidisciplinary heart-team assessment involving interventional cardiologists, cardiac surgeons, cardiac anesthesiologists, clinical engineers, and radiology technicians. Details of the procedural technique have been described previously [9]. The prostheses used during the study period were balloon-expandable SAPIEN-series valves (Edwards Lifesciences, Irvine, CA, USA) and self-expanding Evolut-series valves (Medtronic). The access route (transfemoral, transapical, or trans-subclavian) and valve size were determined based on preprocedural multidetector computed tomography findings and heart team consensus.

Following the procedure, all patients were monitored in a high-dependency care unit for a minimum of 24 h. Those who developed high-degree AV block received temporary pacing support when clinically indicated. Permanent pacing was considered when the attending cardiologist judged that the AV conduction disturbance was unlikely to resolve during the index hospitalization.

### 2.3. Permanent Pacemaker Implantation

The choice between MicraAV and DDD implantation was not based on predefined institutional criteria and was largely at the discretion of the attending physician in consultation with the cardiology team. Clinical considerations included hemodialysis, preservation of venous access, and conditions associated with an increased risk of infection or transvenous device-related complications; patient-specific procedural considerations were also taken into account. Both device types were implanted using standard techniques. For DDD systems, active-fixation atrial and ventricular leads were positioned in the right atrial appendage or interatrial septum and in the right ventricular apex or interventricular septum, respectively. MicraAV deployment initially targeted the mid-right ventricular septum. When pacing or sensing thresholds were suboptimal at this location, or when device deployment was technically challenging, an alternative site—either the right ventricular apex or the high right ventricular septum—was selected.

### 2.4. Electrocardiography and Echocardiographic Parameters

Twelve-lead electrocardiogram (ECG) and transthoracic echocardiographic assessments were obtained at baseline, during the index hospitalization, and at 1, 6, and 12 months of follow-up. Baseline ECGs were assessed for PR interval, QRS duration, QRS axis, and cardiac rhythm.

Echocardiographic assessment included left ventricular ejection fraction (LVEF), left ventricular end-diastolic and end-systolic dimensions (LVDd and LVDs, respectively), mitral regurgitation (MR) and tricuspid regurgitation (TR) severity, and the E/e′ ratio. LVEF was calculated using the biplane disk summation (Simpson) method. Left ventricular dimensions were measured at the level of the mitral valve leaflet tips in the parasternal long-axis view. The severity of MR and TR was graded into four categories—none/trivial, mild, moderate, and severe—following the recommendations of the American Society of Echocardiography guidelines [10]. The E/e′ ratio was calculated by averaging the septal and lateral mitral annular early diastolic velocities (e′) obtained by tissue Doppler imaging and dividing the early mitral inflow velocity (E) by the mean e′ value.

### 2.5. Electrical Performance

Device interrogation was performed at the 1- and 12-month follow-up visits after discharge. The following parameters were recorded: atrial and ventricular sensing amplitudes, pacing threshold, lead impedance, pacing burden, and available device-programming parameters. Ventricular pacing (Vp) burden was expressed as the cumulative ventricular percentage from implantation to the 1-month check (1-month burden) and from the 1-month to the 12-month check (12-month burden). Total Vp burden was defined as the sum of AmVp (ventricular paced beats preceded by detected atrial mechanical contraction) and VpOnly (ventricular paced beats without atrial tracking) for MicraAV, and the sum of ApVp (ventricular paced beats tracking a paced atrial beat) and AsVp (ventricular paced beats tracking a spontaneous atrial contraction) for DDD. For MicraAV, the tracking index was defined as the proportion of AmVp relative to total ventricular paced beats. This device-derived index reflects atrial mechanical tracking but was not considered equivalent to directly measured AV synchrony. Because an analogous tracking index is not generated by conventional DDD devices, no direct between-device comparison of this parameter was performed.

### 2.6. Follow-Up and Clinical Endpoints

Clinical follow-up was scheduled at 1, 6, and 12 months. When an outpatient visit was unavailable, outcome information was collected by telephone or from the patient’s primary care physician.

The prespecified clinical endpoints were all-cause mortality, cardiovascular mortality, heart failure rehospitalization, and device-related complications. Given the exploratory nature and limited sample size of this study, no formal hierarchical testing procedure was applied; all endpoint comparisons should therefore be interpreted as exploratory. Clinical events were defined according to the Valve Academic Research Consortium-3 (VARC-3) criteria [11]. Deaths of unknown cause were classified as cardiovascular mortality, as recommended by these criteria. Heart failure rehospitalization required an unplanned overnight admission for worsening heart failure signs or symptoms accompanied by escalation of medical or mechanical therapy, regardless of the precipitating cause. Device-related complications were defined as any adverse event attributable to the device or implantation procedure, including device infection, pacing threshold elevation, lead dislodgement, lead failure, pericardial effusion, and vascular complications requiring intervention. Pocket or groin hematoma not requiring an invasive procedure was excluded. Pacing threshold elevation was defined as a >2-fold increase from the value recorded at implantation.

Follow-up duration was calculated from the index date to the occurrence of a study endpoint or the last clinical contact. Because follow-up duration differed substantially between the groups, the primary analysis focused on 12-month outcomes with administrative censoring at 365 days.

### 2.7. Statistical Analysis

Continuous variables are summarized as mean ± standard deviation or median with interquartile range (IQR), as appropriate for their distribution. Student’s *t*-test or the Wilcoxon rank-sum test was used for continuous variables, as appropriate, and Fisher’s exact test for categorical variables. All-cause mortality was estimated using the Kaplan–Meier method and compared using an unadjusted Cox proportional hazards model, with hazard ratios (HRs) and 95% confidence intervals (CIs) reported. For nonfatal outcomes, including heart failure rehospitalization and device-related complications, cumulative incidence functions were constructed with all-cause death treated as a competing event, and unadjusted subdistribution hazard ratios (sHRs) with 95% CIs were estimated using Fine–Gray models. Given the small number of clinical events, no multivariable outcome model was fitted. Longitudinal changes in device interrogation and continuous echocardiographic parameters were analyzed using linear mixed-effects models. MR and TR severity were dichotomized as moderate or greater versus less than moderate and analyzed using generalized linear mixed-effects models with a binomial distribution and logit link. The models included group, time, and the group-by-time interaction as fixed effects and patient as a random effect. A two-sided *p* value < 0.05 was considered statistically significant. Analyses were performed using JMP^®^ version 18 (JMP Statistical Discovery LLC., Cary, NC, USA) and R version 4.3.3 (R Foundation for Statistical Computing, Vienna, Austria) for competing-risk analyses.

## 3. Results

### 3.1. Patient Characteristics

Baseline clinical, electrocardiographic, echocardiographic, and procedural characteristics are summarized in Table 1. The DDD group had a higher prevalence of female sex (75.6% vs. 48.3%, *p* = 0.025), whereas cerebrovascular disease was more frequent in the MicraAV group (24.1% vs. 6.7%, *p* = 0.042). Other laboratory, ECG, echocardiographic, and TAVR procedural characteristics were not materially different between groups. Pacemaker implantation time was shorter in the MicraAV group (90 ± 22 min vs. 105 ± 32 min, *p* = 0.036), as was post-implantation hospitalization (4 [3–8] days vs. 8 [5–12] days, *p* < 0.001). Among patients with ventricular leads positioned in the interventricular septum, two received left bundle branch area pacing (LBBAP).

### 3.2. Clinical Outcomes at 12 Months

**Figure 2:**
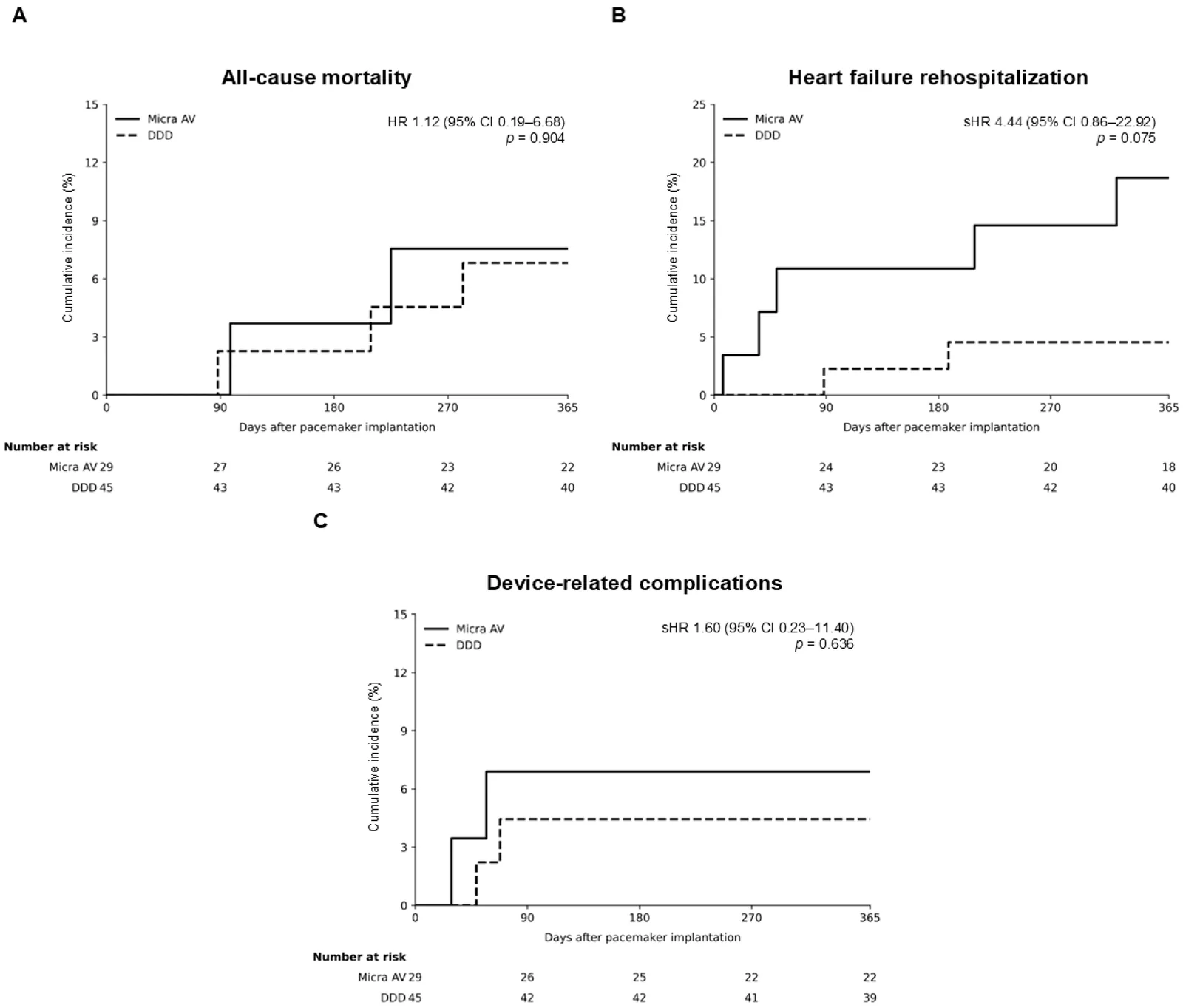
Cumulative event incidence according to pacemaker type during 12-month follow-up. Cumulative event curves for (**A**) all-cause mortality, (**B**) heart failure rehospitalization, and (**C**) device-related complications according to pacemaker type. CI: confidence interval, DDD: dual-chamber transvenous pacemaker, HR: hazard ratio, sHR: subdistribution hazard ratio.

The overall cohort had a median follow-up of 26 months (IQR 13–48), with median follow-up durations of 14 and 40 months in the MicraAV and DDD groups, respectively. The primary analyses were administratively censored at 365 days. All-cause mortality occurred in 6.9% of MicraAV patients and 6.7% of DDD patients (HR, 1.12; 95% CI, 0.19–6.68; *p* = 0.904). All five deaths were cardiovascular in origin (decompensated heart failure, *n* = 3; sudden cardiac death, *n* = 2). Absolute event counts, effect estimates with 95% CIs, and *p* values are presented in Table 2, and the cumulative event curves with numbers at risk are shown in Figure 2.

Heart failure rehospitalization occurred in 5 of 29 patients (17.2%) in the MicraAV group and 2 of 45 patients (4.4%) in the DDD group. In the Fine–Gray competing-risk analysis, with all-cause death treated as a competing event, the unadjusted sHR for MicraAV versus DDD was 4.44 (95% CI, 0.86–22.92; *p* = 0.075). Identified triggers included suspected pacing-induced cardiomyopathy (*n* = 2), inappropriate pacemaker programming (*n* = 1), Takotsubo cardiomyopathy (*n* = 1), radiation-induced pericarditis (*n* = 1), hemodialysis-associated volume overload (*n* = 1), and infection (*n* = 1).

Device-related complications occurred in 2 of 29 patients (6.9%) in the MicraAV group and 2 of 45 patients (4.4%) in the DDD group (sHR, 1.60; 95% CI, 0.23–11.40; *p* = 0.636). Observed complications included pacing threshold elevation in one patient in each group, pacing threshold elevation requiring invasive treatment in one MicraAV patient, and device infection in one DDD patient.

### 3.3. Device Electrical Performance at Follow-Up

**Figure 3:**
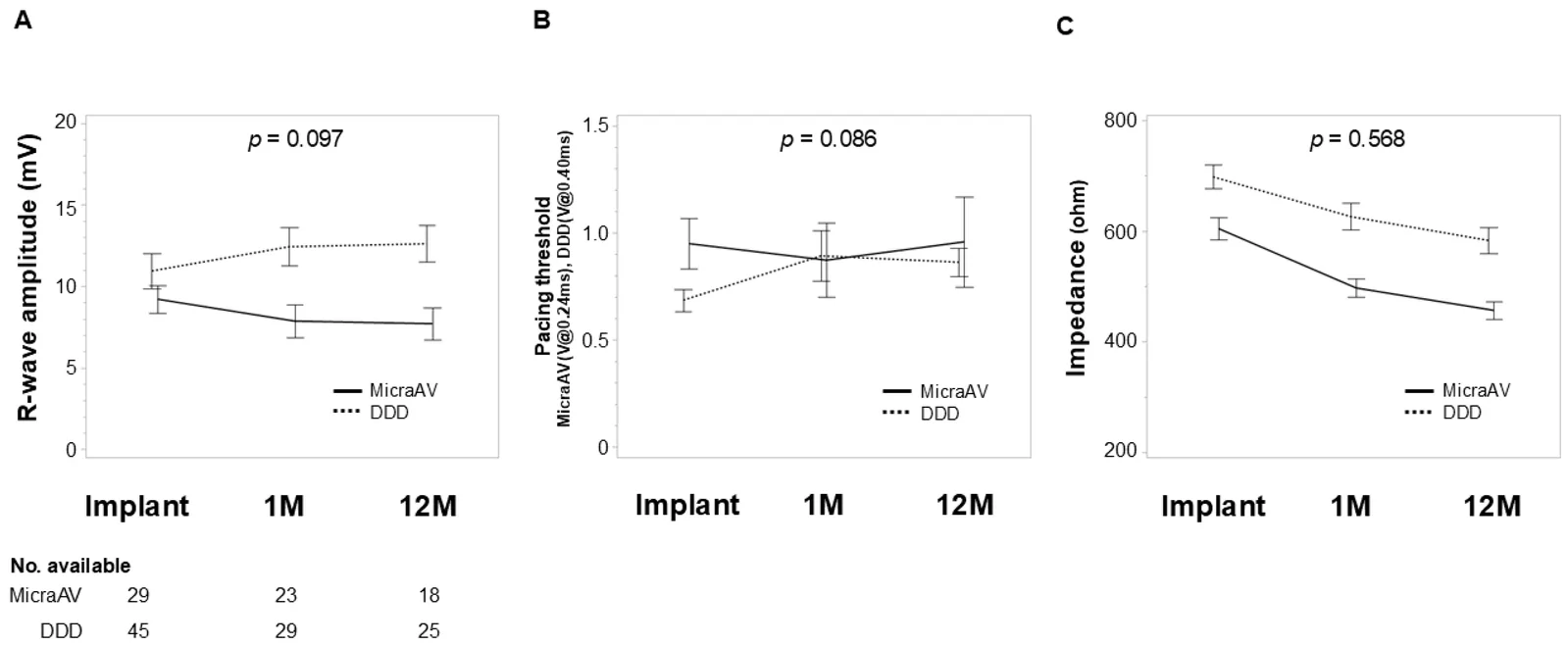
Longitudinal changes in device interrogation parameters during follow-up. Longitudinal changes in (**A**) R-wave amplitude, (**B**) pacing threshold, and (**C**) lead impedance in the MicraAV and DDD groups. Data are presented as mean ± standard error. The numbers of patients with available device interrogation data at each time point are shown below the x-axis in panel (**A**) and apply to all panels. *p* values represent group-by-time interaction derived from linear mixed-effects model analyses. DDD: dual-chamber transvenous pacemaker, M: month.

Pacemaker programming and pacing characteristics during follow-up are summarized in Table 3. A lower rate limit of 50 bpm was more frequently programmed in the MicraAV group, whereas 60 bpm was more frequently programmed in the DDD group. Ventricular pacing burden, assessed at censoring or the end of the study period, was 87.5% [IQR, 50.7–95.4] in the MicraAV group and 96.3% [IQR, 5.099.7] in the DDD group (*p* = 0.374), and the median MicraAV tracking index was 88.4% [IQR, 66.4–95.1]. During follow-up, pacing mode was changed from VDD to VVI in one MicraAV patient because of the development of atrial fibrillation, with no mode changes in the remaining patients. Longitudinal device interrogation showed generally stable R-wave amplitude, pacing threshold, and impedance, with no significant group-by-time interactions (*p* = 0.097, 0.086, and 0.568, respectively; Figure 3).

### 3.4. Serial Echocardiographic Changes Following Pacemaker Implantation

**Figure 4:**
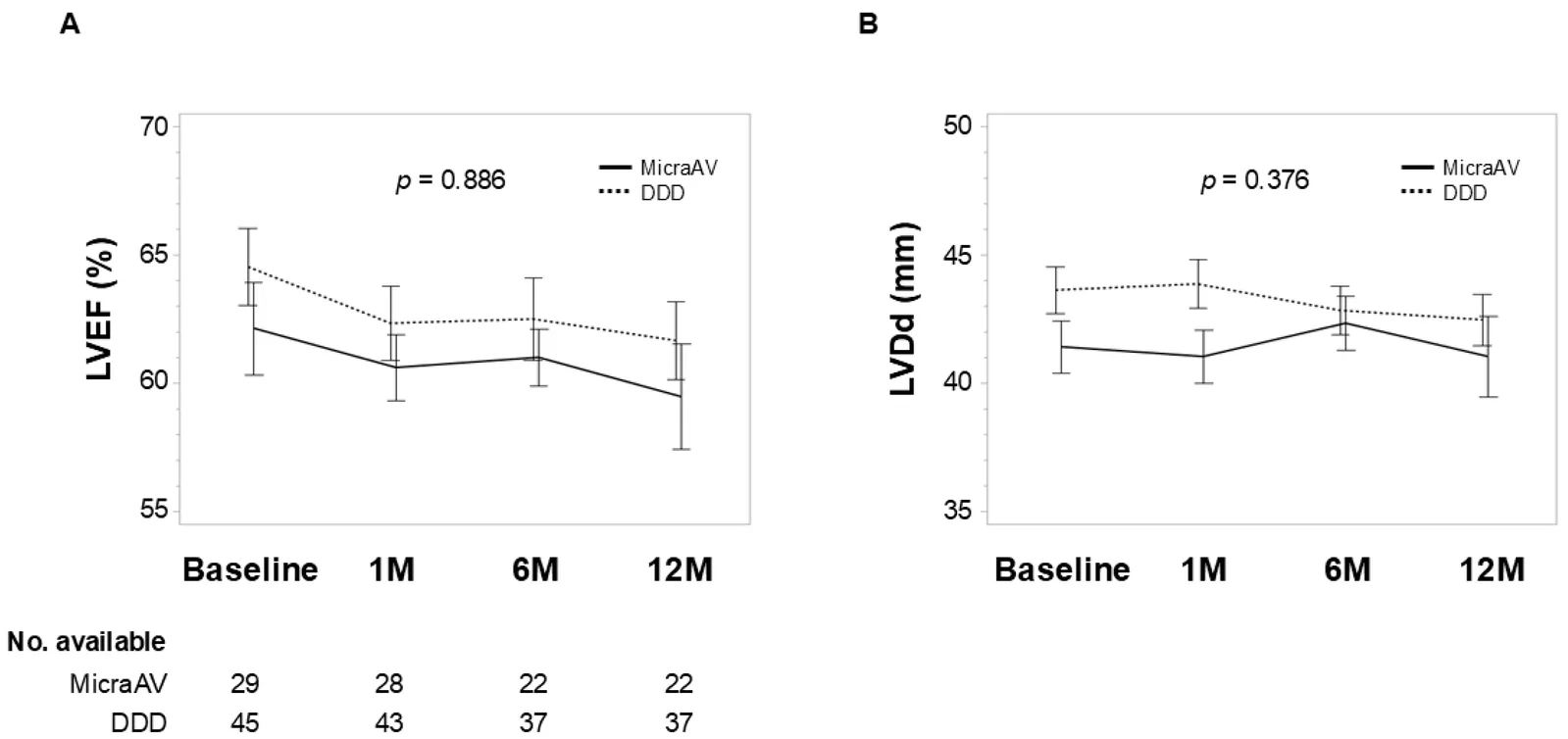

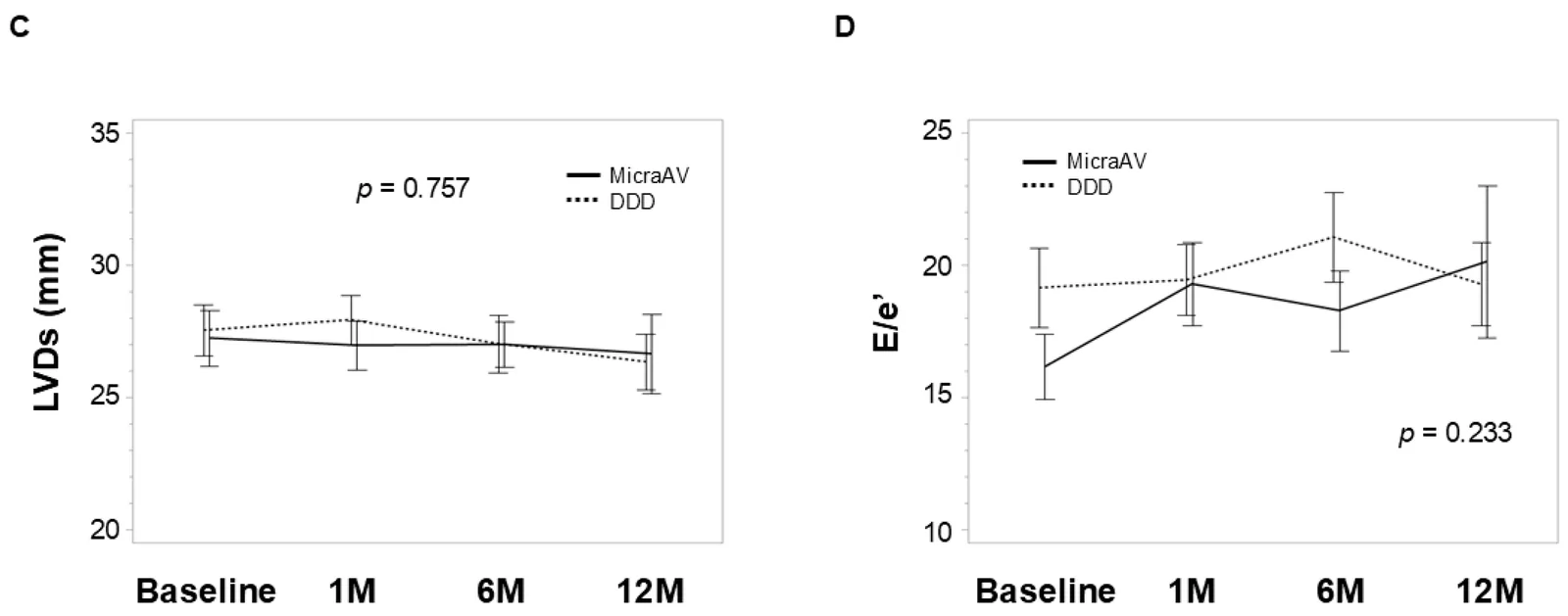
Longitudinal changes in echocardiographic parameters during follow-up. Longitudinal changes in (**A**) left ventricular ejection fraction, (**B**) left ventricular end-diastolic dimension, (**C**) left ventricular end-systolic dimension, and (**D**) E/e′ ratio in the MicraAV and DDD groups. Data are presented as mean ± standard error. The numbers of patients with available echocardiographic data at each time point are shown below the x-axis in panel A and apply to all panels. *p* values represent group-by-time interactions derived from linear mixed-effects model analyses. DDD: dual-chamber transvenous pacemaker, LVDd: left ventricular end-diastolic dimension, LVDs: left ventricular end-systolic dimension, LVEF: left ventricular ejection fraction, M: month.

**Figure 5:**
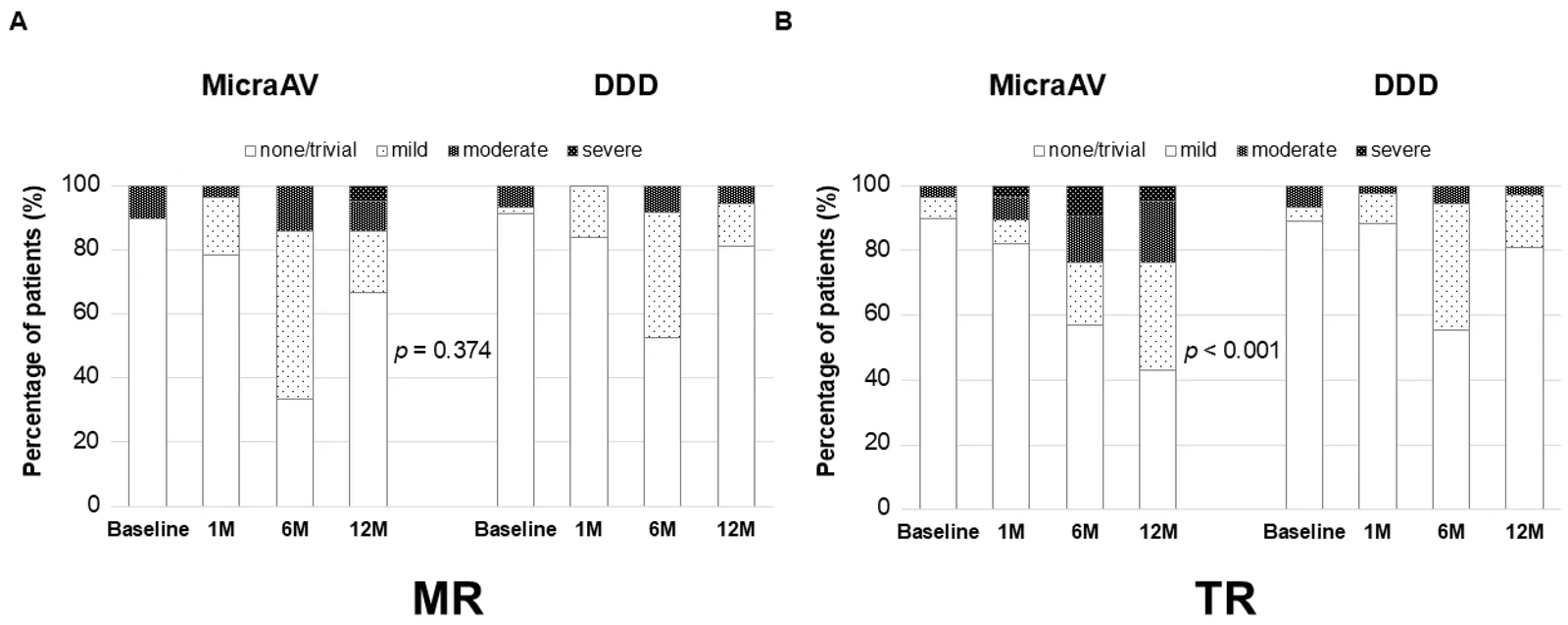
Longitudinal changes in mitral and tricuspid regurgitation severity following pacemaker implantation. Longitudinal distributions of (**A**) mitral regurgitation (MR) and (**B**) tricuspid regurgitation (TR) severity in the MicraAV and DDD groups. For statistical analysis, MR and TR severity were dichotomized as moderate or greater versus less than moderate. *p* values represent group-by-time interactions for the dichotomized outcomes. The numbers of patients with available echocardiographic data at each time point are the same as those shown in Figure 4. DDD: dual-chamber transvenous pacemaker, M: month, MR: mitral regurgitation, TR: tricuspid regurgitation.

Longitudinal changes in LVEF, LVDd, LVDs, and E/e′ ratio during follow-up are shown in Figure 4. Linear mixed-effects models revealed no significant group-by-time interaction for these continuous parameters (*p* = 0.886, 0.376, 0.757, and 0.233, respectively). Figure 5 illustrates longitudinal MR and TR severity. The analysis did not demonstrate a significant between-group difference in the temporal pattern of moderate-or-greater MR (*p* = 0.374), whereas TR severity increased over time within the MicraAV group (*p* < 0.001).

## 4. Discussion

### 4.1. Main Findings

In this single-center exploratory study, MicraAV and DDD after TAVR were associated with imprecisely estimated 12-month clinical outcomes because of the small number of events. Heart failure rehospitalization occurred more frequently in the MicraAV group, whereas mortality and device-related complication estimates had wide confidence intervals spanning potentially important benefit and harm. Longitudinal device parameters and ventricular function showed no significant group-by-time interaction, while TR worsened over time within the MicraAV group. The incremental contribution of this study is its direct comparison of MicraAV-specific outcomes with DDD after TAVR, including device performance and echocardiographic changes—information that is not captured by earlier TAVR studies dominated by ventricular-only leadless pacing.

### 4.2. Mortality

Previous observational studies have generally reported acceptable mortality outcomes with leadless pacing, although direct comparisons between MicraAV and DDD systems remain limited and are susceptible to confounding by differences in patient selection [12,13]. In the present cohort, the HR for all-cause mortality was 1.12, but the 95% CI was wide (0.19–6.68), reflecting the very small number of deaths. Accordingly, the absence of a statistically significant difference should not be interpreted as evidence of equivalence between MicraAV and DDD.

Given that TAVR recipients are predominantly elderly with multiple comorbidities, non-cardiac causes of death might be expected to predominate; however, of the five deaths observed, all were cardiovascular in origin, including decompensated heart failure (*n* = 3) and sudden death (*n* = 2). Notably, a previous report from our group identified a higher rate of sudden cardiac death in the MicraVR group following TAVR [8], and whether a similar trend exists with MicraAV remains to be determined. Larger studies with extended follow-up are needed to draw definitive conclusions.

### 4.3. Heart Failure Rehospitalization

Heart failure rehospitalization occurred more frequently in the MicraAV group, with an unadjusted sHR of 4.44; however, the 95% CI was wide (0.86–22.92), reflecting only seven events. A historical concern with ventricular-only leadless pacing is loss of AV synchrony, which may adversely affect hemodynamics and heart failure risk [5,6,8]. MicraAV was developed to mitigate this limitation by using atrial mechanical sensing to facilitate AV-synchronous ventricular pacing [7]. Nevertheless, atrial mechanical tracking varies among patients and with activity, and a device-derived tracking index is not equivalent to direct measurement of AV synchrony [14]. The observed higher rate of heart failure rehospitalization in the MicraAV group may reflect device-related factors, differences in ventricular activation, patient selection, or unmeasured confounding. However, given the very small number of heart failure rehospitalization events, the present study was not adequately powered to distinguish among these potential mechanisms.

Conduction system pacing, particularly LBBAP, has shown favorable clinical and electrical outcomes after TAVR [15]. Two patients in the DDD group underwent LBBAP, which could potentially influence heart failure outcomes. However, excluding these patients yielded a similar, although imprecise, estimate for heart failure rehospitalization (sHR, 4.24; 95% CI, 0.82–21.87; *p* = 0.084), suggesting that the primary finding was not driven solely by LBBAP.

### 4.4. Device-Related Complications

Leadless pacemakers avoid transvenous leads and a subcutaneous generator pocket and have been associated with lower rates of several device-related complications and system revisions in large observational cohorts [16,17]. Contemporary MicraAV data similarly suggest lower major complication and reintervention rates than DDD, although residual confounding remains possible [12,13]. These safety data are relevant to TAVR recipients, who are typically older and may have substantial comorbidity or an increased risk of infection. In the present study, no device infection occurred in the MicraAV group, and procedural time and post-implantation hospitalization were shorter than with DDD.

Nevertheless, only four device-related complications occurred in the present cohort, and the sHR estimate was highly imprecise (1.60; 95% CI, 0.23–11.40; *p* = 0.636). Therefore, these data cannot establish comparative safety or equivalence between MicraAV and DDD. The observed procedural advantages and absence of infection in MicraAV recipients should be interpreted as descriptive findings that require confirmation in larger comparative studies.

### 4.5. Device Electrical and Echocardiographic Performance

Electrical parameters remained generally stable during follow-up, although one MicraAV patient developed a marked pacing-threshold increase requiring invasive revision. Because leadless and transvenous systems differ substantially in electrode design, fixation, and electrical characteristics, direct numerical comparisons should be interpreted cautiously rather than as evidence of equivalent electrical performance. The low implantation impedance (510 Ω) in the patient requiring revision is notable because lower implantation impedance has been associated with subsequent threshold deterioration [18].

Echocardiographic parameters were generally stable in both groups, whereas TR severity increased over time within the MicraAV group. Because MicraAV does not traverse the tricuspid valve, a direct lead-related mechanism is unlikely. One possible explanation is imperfect AV coordination in some patients; however, the tracking index is only a device-derived marker of atrial mechanical tracking and does not directly quantify AV synchrony. Moreover, contemporary data have reported a low prevalence of new-onset severe TR after leadless pacemaker implantation [19]. Accordingly, the association observed in our cohort should be regarded as hypothesis-generating rather than causal. Larger studies using direct or validated measures of AV synchrony and appropriate ordinal longitudinal models for TR severity are needed.

### 4.6. Study Limitation

Several limitations of the present study should be acknowledged. First, this was a small, retrospective, single-center observational study with few clinical events, resulting in limited statistical power and wide confidence intervals. Second, device selection was nonrandomized and largely at the discretion of the treating physician, introducing potential selection bias and residual confounding. Third, the study spanned 2016–2025, whereas MicraAV became available only during the latter part of the study, introducing potential treatment-era confounding. Finally, follow-up and device-interrogation data were incomplete for some patients, and detailed AV synchrony measurements and programming parameters were not uniformly available.

## 5. Conclusions

In this small observational cohort of patients requiring permanent pacing for AV block after TAVR, heart failure rehospitalization occurred more frequently in the MicraAV group, whereas mortality and device-related complications were infrequent in both groups. These exploratory findings require confirmation in larger prospective studies.

## Figures and Tables

**Table 1:** Baseline and procedural characteristics.

	MicraAV *n* = 29	DDD *n* = 45	*p* Value
**Clinical characteristics:**			
Age, yrs	84 ± 5	83 ± 5	0.791
Women, *n* (%)	14 (48.3)	34 (75.6)	0.025
BMI, kg/m^2^	22 ± 3	23 ± 4	0.449
NYHA ≥ III, *n* (%)	9 (31.0)	12 (26.7)	0.793
STS score, %	6.3 ± 4.4	6.5 ± 4.0	0.822
Hypertension, *n* (%)	20 (69.0)	36 (80.0)	0.406
Diabetes mellitus, *n* (%)	13 (44.8)	14 (31.1)	0.323
COPD, *n* (%)	4 (13.8)	3 (6.7)	0.422
Dialysis, *n* (%)	4 (13.8)	3 (6.7)	0.422
History of PAF, *n* (%)	5 (17.2)	6 (13.3)	0.742
History of CAD, *n* (%)	8 (27.6)	12 (26.7)	1.000
History of CVD, *n* (%)	7 (24.1)	3 (6.7)	0.042
Hemoglobin, g/dL	11.6 ± 1.5	11.2 ± 1.4	0.219
eGFR, mL/min/1.73 m^2^	45 ± 22	46 ± 20	0.807
Serum albumin, g/dL	3.7 ± 0.5	3.6 ± 0.6	0.582
NT-proBNP, pg/mL	809 (349–2751)	1128 (518–2480)	0.463
**ECG characteristics:**			
PR interval, ms	193 ± 49	188 ± 38	0.667
QRS duration, ms	128 ± 29	126 ± 27	0.766
QRS axis, °	10 ± 61	21 ± 56	0.451
Paced QRS duration after PMI, ms	163 ± 17	159 ± 19	0.309
**Echocardiographic parameters:**			
LVEF, %	62 ± 10	65 ± 10	0.312
LVDd, mm	41 ± 6	44 ± 6	0.113
MR ≥ moderate, *n* (%)	3 (10.3)	3 (6.7)	0.674
TR ≥ moderate, *n* (%)	1 (3.5)	3 (6.7)	1.000
E/e′ ratio	16.2 ± 6.6	19.1 ± 9.9	0.128
**Procedural parameters in TAVR:**			
Transfemoral approach, *n* (%):	26 (89.7)	43 (95.6)	0.374
Prosthesis size ≥ 29 mm, *n* (%)	2 (6.9)	2 (4.4)	0.642
Self-expandable valve, *n* (%)	10 (34.5)	13 (28.9)	0.618
**Procedural parameters in PMI:**			
Implantation timing after TAVR, days	4 (1–5)	5 (2–8)	0.056
Procedural time, min	90 ± 22	105 ± 32	0.036
RV pacing site in apex, *n* (%)	10 (34.5)	26 (57.8)	0.060
Hospitalization after PMI, days	4 (3–8)	8 (5–12)	<0.001

Data are expressed as *n* (%) for categorical variables and as mean ± standard deviation or median (interquartile range) for continuous variables. BMI: body mass index, CAD: coronary artery disease, COPD: chronic obstructive pulmonary disease, CVD: cerebrovascular disease, DDD: dual-chamber transvenous pacemakers, ECG: electrocardiogram, eGFR: estimated glomerular filtration rate, LVDd: left ventricular end-diastolic diameter, LVEF: left ventricular ejection fraction, MR: mitral regurgitation, NT-proBNP: N-terminal pro-brain natriuretic peptide, NYHA: New York Heart Association, PAF: paroxysmal atrial fibrillation, PMI: pacemaker implantation, RV: right ventricular, STS: Society of Thoracic Surgeons, TAVR: transcatheter aortic valve replacement, TR: tricuspid regurgitation.

**Table 2:** Twelve-month clinical outcomes and effect estimates.

Outcome	MicraAV (*n* = 29) Observed Events (% ^a^)	DDD (*n* = 45) Observed Events (% ^a^)	Effect Estimates ^b^ (95% CI)	*p* Value
All-cause mortality	2 (6.9)	3 (6.7)	HR 1.12 (0.19–6.68)	0.904
Cardiovascular mortality	2 (6.9)	3 (6.7)	HR 1.12 (0.19–6.68)	0.904
Heat failure rehospitalization	5 (17.2)	2 (4.4)	sHR 4.44 (0.86–22.92)	0.075
Device-related complications	2 (6.9)	2 (4.4)	sHR 1.60 (0.23–11.40)	0.636

CI: confidence interval, DDD: dual-chamber pacemaker, HR: hazard ratio, sHR: subdistribution hazard ratio. ^a^ Raw percentage defined as number of events divided by number of patients included in the group. ^b^ HRs were estimated using univariable Cox proportional hazards models. For nonfatal outcomes, sHRs were estimated using Fine–Gray competing-risk models, with all-cause death treated as a competing event. DDD was used as the reference group. All effect estimates are unadjusted.

**Table 3:** Pacemaker programming and pacing characteristics during follow-up.

	MicraAV *n* = 29	DDD *n* = 45	*p* Value
Mode change to VVI during follow-up, *n* (%)	1 (3.4)	0 (0.0)	0.392
Lower rate limit, bpm, *n* (%)			<0.001
≤40	0 (0.0)	0 (0.0)	
50	19 (65.5)	4 (8.9)	
60	10 (34.5)	35 (77.8)	
≥70	0 (0.0)	6 (13.3)	
Ventricular pacing percentage ^a^, %	87.5 (50.7–95.4)	96.3 (5.0–99.7)	0.374
Tracking index ^a,b^, %	88.4 (66.4–95.1)	–	–

Values are *n* (%) or median (interquartile range), as appropriate. DDD: dual-chamber pacemaker. ^a^ Device interrogation data were available for 24 Micra AV patients and 35 DDD patients. ^b^ Tracking index was available only for patients with Micra AV.

## Data Availability

The data related to this article are not available due to privacy or ethical restrictions.

## Abbreviations

The following abbreviations are used in this manuscript:

AmVp	Ventricular paced beats tracking atrial mechanical contraction
ApVp	Ventricular paced beats tracking a paced atrial beat
AsVp	Ventricular paced beats tracking a spontaneous atrial contraction
AV	Atrioventricular
DDD	Dual-chamber transvenous pacemaker
ECG	Electrocardiogram
HR	Hazard ratio
IQR	Interquartile range
LVDd	Left ventricular end-diastolic dimension
LVDs	Left ventricular end-systolic dimension
LVEF	Left ventricular ejection fraction
MR	Mitral regurgitation
sHR	Subdistribution hazard ratio
TAVR	Transcatheter aortic valve replacement
TR	Tricuspid regurgitation
Vp	Ventricular pacing
VpOnly	Ventricular paced beats without atrial tracking
